# Antidepressants in the Management of Premenstrual Dysphoric Disorder: Evidence and Insights

**DOI:** 10.1192/j.eurpsy.2026.11771

**Published:** 2026-07-31

**Authors:** I. Mosbah, H. Ghabi, R. kammoun, A. karmous, H. nefzi, M. karoui, F. Ellouze

**Affiliations:** 1 Department of Psychiatry G, Razi Hospital, Manouba; 2 El Manar University, Faculty of Medicine of Tunis, Tunis; 3 Department of Psychiatry A, Razi Hospital, Manouba, Tunisia

## Abstract

**Introduction:**

Premenstrual dysphoric disorder (PMDD) was officially recognized in the DSM-5 in 2013. This disorder, which can be particularly debilitating, affects patients’ personal, social, familial, and professional functioning. The prevalence of PMDD ranges from 1.8% to 5.8% among women of reproductive age. Subclinical forms are very common, affecting 50% to 90% of women reporting premenstrual symptoms. Despite this high frequency, more than half of affected women do not receive an adequate diagnosis and remain undertreated. These findings highlight that premenstrual cycle–related symptoms represent one of the most frequent psychiatric and gynecological disorders, yet remain underdiagnosed and insufficiently treated.

**Objectives:**

The aim of our study was to review the efficacy of antidepressants in PMDD and their prescription modalities.

**Methods:**

A literature search was conducted between January 2005 and January 2025 in the APA PsycInfo, Scopus, and PubMed databases. After an initial selection based on keywords, full-text screening identified nine studies relevant for the final analysis.

**Results:**

Antidepressants appear to be the first-line treatment for PMDD, with selective serotonin reuptake inhibitors (SSRIs) showing particularly strong efficacy. These findings support the current etiological hypothesis that natural hormonal fluctuations exert an adverse effect on certain neurotransmitters. SSRIs such as sertraline (50–150 mg/day), fluoxetine (10–20 mg/day), escitalopram (10–20 mg/day), and paroxetine (12.5–25 mg/day) are considered the treatments of choice. Unlike in other mood disorders, SSRIs in PMDD demonstrate a rapid onset of therapeutic action, manifesting within hours to days. This explains why an intermittent regimen (restricted to the luteal phase, i.e., from ovulation to the onset of menstruation) is a feasible option.

**Conclusions:**

To optimize the evaluation of PMDD treatment efficacy, future studies should be standardized with respect to study design, treatment dosage, and assessment methods.

**Disclosure of Interest:**

None Declared

